# Comparison of hematological aspects among children with Malaria and healthy children 

**Published:** 2015-04-20

**Authors:** A Fattahi Bafghi, A Hashemi, S Abolhasanizadeh

**Affiliations:** 1 1.Associate Professor, Medical Parasitology and Mycology Department, school of Medicine, Shahid Sadoughi University of Medical Sciences and Health Services, Yazd, Iran.; 2 2.Associate professor, Pediatric Hematology & Oncology Department and Editor in chief Iranian Journal of Pediatric Hematology & Oncology Shahid Sadoughi University of Medical Sciences, Yazd, Iran.; 3 Msc Microbiology Student, microbiology Department, The international campus, Shahid Sadoughi University of Medical Sciences and Health Services, Yazd, Iran.

**Keywords:** Children, Falciparum, Hematological aspects, Malaria, Plasmodium, Vivax

## Abstract

**Background:**

Malaria is an acute and chronic illness characterized by paroxysms of fever, chills, sweating, fatigue, anemia, and splenomegaly. Most malarial deaths occur in infants and young children.Anemia in malaria has diverse pathophysiologic mechanisms such as direct invasion of Red cells, In the following, we presented comparison of hematological aspects: children with Malaria and healthy children.

**Materials and Methods:**

This was a lab trial study. Patients were referred and admitted to the pathobiology laboratory along with physical examination. Then, they underwent a complete blood count and the result of complete blood count was compared with healthy person in the same age. Out of 30 patients, with equally

falciparum, vivax, and healthy .The hematological examination was performed. Finally, the data was analyzed using SPSS version 19 software.

**Results:**

The levels of HGB (P=0.001), HCT (P=0.001), MCV (P= 0.001), MCH (P=0.001), WBC (P=0.001), and Plt (P= 0.02) decreased significantly in children with falciparum and vivax malaria compared to healthy controls. The levels of RBC (P=0.49) increased significantly in children with falciparum and vivax malaria compared to controls. Blood culture at two times. To investigate malaria; blood smears taken after microscopic study of Plasmodium falciparum ring was observed.

**Conclusion:**

Malaria is a multisystem disorder which can lead to many diseases. Physicians, especially those in endemic areas, should be aware of the varied manifestations and maintain in a high index of suspicion for the disease in order to that diagnose and treat timely and, morbidity and mortality.

## Introduction

Malaria is an acute and chronic illness characterized by paroxysms of fever, chills, sweating, fatigue, anemia, and splenomegaly. Most malarial deaths occur in infants and young children. In spite of intensive worldwide efforts to reduce its transmission, malaria remains the most serious and widespread protozoal infection of humans. Over 40% of the world’s populations are at risk of malaria, which is endemic in 91 countries, mostly developing. Most of the systemic complications from malaria results from heavy parasitemia. Hematological changes are the most common complications encountered in malaria and play major role in the fatality. Plasmodium falciparum causes the most severe form of malaria and is associated with more intense parasitemia. It is one of the most prevalent human infections in the world. It is estimated that 300-500 million cases and 1.5-2.7 million deaths occur each year. Mortality rate is usually high (20%) in severe malaria (parasitemia >5%). Over 40% of world population live in malaria endemic area including Southeast Asia, India, Africa and areas of Middle East, Central and South America [1, 2 and 3]. Iran is in the elimination phase of malaria, in other words in the following years we do expect to find much fewer cases in Iran. However, it is not easy to predict the slope of its decreasing without a deep exploration of the situation. For such a purpose, we have to be familiar with the key concepts of the forecasting and trend analysis. Trend analysis means the exploring of temporal variations of a variable such as the number of malaria cases. For such an analysis, the scope of exploration might be short (few years), intermediate (around one decade), or long term. In addition, we have different shapes of temporal variations such as cyclic (such as seasonal), linear, or complex trends. Moreover, we may assess the geographical variation of available. Usually the technical word of spatial analysis is used, and maps are the best way to illustrate the distribution of the variable of interest [4, 5, 6, and 7]. The clinical outcome of a malarial infection in a child depends on many: parasite, host, geographic and social factors. Iran has achieved an 85 percent decrease in reported malaria cases between 2000 and 2010, from 12,294 cases to 1,847 cases, and is categorized in the elimination phase by the World Health Organization. Iran has reported less than five deaths due to malaria annually since 1999. In 2010, only three percent of the population was considered at-risk of malaria transmission; nearly 40 percent of all malaria cases were imported, and approximately 85 percent of the total was due to Plasmodium vivax with the remainder due to Plasmodium falciparum. Eight vectors are responsible for malaria transmission in Iran and the four dominant vectors are Anopheles superpictus, An. sacharovi, An. stephensi, and the An. fluviatilis species complex. Nearly all malaria transmission occurs in the south-eastern areas of the country near its borders with Afghanistan and Pakistan, and transmission peaks in the summer months of August and September.3 In 2010, more than 90 percent of malaria cases were reported in the region of Iran in three provinces, Hormozgan, Kerman, and Sistan and Baluchistan. All of which are near the border with Pakistan. A majority of the population in these provinces live in rural areas and have the least access to primary health care services, the lowest literacy levels, and the highest maternal and child mortality rates. The Iranian national malaria strategic plan has set goals to reduce local malaria transmission and continue to prevent malaria deaths in targeted districts though early diagnosis, prompt and effective treatment, and vector control through indoor residual spraying (IRS) and distribution of long-lasting insecticide-treated nets, in addition to establishing a malaria early warning system for controlling malaria epidemics. Through these efforts, Iran is aiming to eliminate P. falciparum by 2015 and to become malaria-free by 2025 [8, 9, 10, 11 and 12]. In the following, we presented comparison of hematological aspects: children with falciparum & vivax malaria and healthy.

## Materials and Methods

This was a lab trial study. Patients were referred and admitted to the pathobiology laboratory along with physical examination. Out of 30 patients, with equally falciparum, vivax, and healthy.Then, they underwent a complete blood count (CBC) Based on the following procedures: The methods used to derive CBC parameters are based on the Beckman Coulter measurements of these three parameters. Result of method of counting and sizing, in combination with an automatic diluting and mixing device for sample processing, and a single beam photometer for. The WBC differential uses VCS technology. Analysis and classification of WBCs use three simultaneous measurements of individual cell volume (V), high frequency conductivity (C), and laser light scatter (S). The scatter gram plots the cells based upon the complete blood count was compared with healthy person in the same age. The hematological was performed for children with Malaria and as well as healthy children. Serum levels of Hemoglobin, Hematocrit, HGB, MCV, MCH, RBC, WBC and, platelet were measured.

**Statistical Analysis**

The data was analyzed using SPSS version 19 statistical software with appropriate statistical methods. Differences were considered significant at P-values of less than 0.05. Informed consent was taken from patients and parents.

## Results

Of the total 30 cases of malaria falciparum, vivax and healthy choices were equally, the levels of HGB (P=0.001), HCT (P=0.001), MCV (P= 0.001), MCH (P=0.001), WBC (P=0.001), and Plt (P= 0.02) decreased significantly in children with falciparum and vivax malaria compared to healthy controls. The levels of RBC (P=0.49) increased significantly in children with falciparum and vivax malaria compared to controls. Blood culture at two times. To investigate malaria; blood smears were taken after microscopic study of Plasmodium falciparum ring was observed (Figure. 2). Fortunately, Plasmodium falciparum chloroquine-sensitive and the patient were discharged in good condition and pancytopenias were also on hand (Table I).

**Table I:** Comparison of hematological aspects: children with falciparum, vivax malaria and healthy

Group	HGB g/dl	HCT %	MCV fl	MCH pg	RBC µl	*WBC* µl	Plt µl
falciparum Patients	10.21±0.62 MD=10.25	34.92±1.4 MD=35.55	72.73±4.75 MD=71.55	19.9±0.54 MD=19.93	4.84±0.12 MD=4.85	5.09±0.35 MD=5.25	263.8±59.64 MD=280.5
vivax patients	12.2±0.75 MD=12.25	37.5±1.62 MD=37.75	73.48±0.84 MD=73.35	23.61±0.76 MD=23.25	4.8±0.52 MD=4.79	11.5±0.41 MD=11.55	408.2±328.73 MD=328
Healthy	26.44±40.3 MD=13.65	41.31±3.75 MD=40.75	84.66±3.32 MD=85.5	27.88±0.91 MD=27.9	5.02±0.53 MD=4.92	8.81±3.36 MD=8.35	317.1±92.15 MD=302
Test	Kruskal-Wallis Test	ANOVA	ANOVA	ANOVA	ANOVA	ANOVA	Kruskal-Wallis Test
P-value	<0.001	<0.001	<0.001	<0.001	0.49	<0.001	0.002

## Discussion

Hematological abnormalities are thought-outed a hallmark of malaria, and reported to be most in Plasmodium falciparum infection, probably as a result of the higher levels of parasitemia found in these patients. Malaria is a multisystem disorder which can cause many diseases. Physicians, especially those in endemic areas, should be aware of the varied manifestations and maintain a high index of suspicion for the disease in order to diagnose and treat timely and morbidity and mortality minimized. Hemophagocytic syndrome and erythrophagocytosis, dyserythropoirsis, immune haemolysis and cytokine dysregulation Anemia of chronic disorder is characterized by moderate to mild normocytic normochromic anemia along with microcytic hypochromic cells. Evaluation of Malaria infection 

especially in malaria- borne areas is highly recommended for consideration and further therapy [13 and 14]. Malaria is a common cause of fever in tropical countries and First Symptoms nonspecific and includes headache, fatigue, myalgia, abdominal pain, and fever and sometimes causes arthralgia, diarrhea, and chest pain. Nausea, vomiting, and orthostatic hypotension are also common [15]. The hematological involvement as medicinal and mild leukocytosis, thrombocytopenia, and sometimes it is hematological changes are common complications encountered in severe malaria [16]. Our finding is in consistent with previous reports. In this study, we observed significantly lower values of HGB/dl, HTC %, MCV fl and MCH µl WBC µl, and Plt µl among malaria-infected children compared to the health children. Our findings are also in consistent with previous reports which observed a higher incidence of anaemias among parasitized children compared to controls [17 and 18]. Similarly, a previous report indicates that there is imbalance in RBC surface markers such as CR1 [19]. Anemia is due to red blood cells and removal rate Erythropoisis by spleen cells and ineffective, but the causes of pancytopenia and Hemophagocytosis does not occur. Always there is a direct correlation between parasitemia and thrombocytopenia. Diagnosis is based on the existence of malaria parasites in peripheral blood smear that is done asexual forms (thin & thick smear Platelet count, CRP and ESR thick). In laboratory studies, anemia was common Normochromic normocytic be disrupted. PTT and PT 100000 reduced. In severe infections, treatment of non-falciparum malaria, chloroquine is the drug of choice for severe malaria but not to Chloroquine trusted. It must be quinine or Kynydyn or Fnsydar (sulfadoxine + Prymtamyn) can be used [20, 21 and 22].

## Conclusion

In children, Plasmodium parasitaemia has a significant impact on the packed cell Volume, hemoglobin, and platelet. Preventative strategies including intermittent preventative, regular chemoprophylaxis, Treatment with antimalarials, provision of iron supplementation, and insecticide-treated bed nets should be implemented urgently to prevent the negative impact of malaria Parasitaemia on the hematological parameters of children in the area. There is a necessity for community and peer-based Education initiatives to strengthen the malaria Prevention programme by educating parents on the benefits of effective environmental sanitation to destroy the breeding sites of Anopheles mosquito –the vector of malaria and awareness. 

## References

[1] Ali Fattahi Bafghi, Seyed Hossein Shahcheraghi, Sedigheh Nematollahi Comparison of hematological aspects: Visceral Leishmaniasis and healthy children. http://www.Tropicalparasitology.org.

[2] Syyed Ahmed Zaki, Preeti Shanbag (2011). Atypical manifestations of malaria. Research and Reports in Tropical Medicine.

[3] Soraya Kaewpitoon, Rattana Rujiragul, Niwatchai Namwichaisirikul, Seekaow Churproong, Naporn Ueng-arporn, Likit Matrakool, Natthawut Kaewpitoon (2012). Malaria in Thailand 2007-2011. Srinagarind Med J.

[4] Maksudur Rahman, Hossain Shahid Kamrul Alam, Abu Tayeb, Probir Kumar Sarker, Tahera Nazreen, Akhand Tanzih Sultana (2011). Malaria - An update. DS (Child) H J.

[5] Kalyan Kumar Kuthala, Sowjanya Meka, Sunita Kanikaram (2013). A Study on Course of Infection and Haematological Changes in falciparum-Infected in Comparison with Artemisinin(s)-Treated Mice. Malar Res Treat.

[6] Syyed Ahmed Zaki, Preeti Shanbag (2011). Atypical manifestations of malaria. Research and Reports in Tropical Medicine.

[7] Ali Fttahi Bafghi, Sayyed Ali Reza Pourmazar, Farimah Shamsi ( 2013). Five-Year Status of Malaria (a Disease Causing Anemia) in Yazd, 2008-2012. Iranian Journal of Pediatric Hematology Oncology.

[8] Mohsen Rezaei Hemami, Ali Akbari Sari, Ahmad Raeisi, Hassan Vatandoost, Reza Majdzadeh (2013). Malaria Elimination in Iran, Importance and Challenges. Journal List Int J Prev Med.

[9] Sinka, M.E (2010). The dominant Anopheles vectors of human malaria in Africa, Europe and the Middle East: occurrence data, distribution maps and bionomic precis. Parasit Vectors.

[10] Haghdoost AA, Alexander N, Cox J (2008). Modelling of malaria temporal variations in Iran. Tropical Medicine and International Health.

[11] (2010). The Global Fund to Fight AIDS, Tuberculosis and Malaria. Round 10 proposals: Elimination of Falciparum malaria in priority areas in the Islamic Republic of Iran.

[12] (2008). The Global Fund to Fight AIDS, Tuberculosis and Malaria. Round 7: Malaria Intensified Control in High Burden Provinces of South Eastern Iran.

[13] Raeisi, A (2012). Malaria elimination in IR Iran, achievements and challenges in IR Iran.

[14] Leonardo Chianura, Isabella Corinna Errante, Giovanna Travi, Roberto Rossotti, Massimo Puoti (2012). Hyperglycemia in Severe Falciparum Malaria: A Case Report. Case Reports in Critical Care.

[15] S. A. Zaki, P. Shanbag (2011). Atypical manifestations of malaria. Research and Reports in Tropical Medicine.

[16] Blanc B, Finch CA, Hall berg L (1968). Nutritional anaemias. Report of a WHO Scientific Group. WHO Tech Rep Ser.

[17] Maina RN, Douglas Walsh, Charla Gaddy, Gordon Hongo, John Waituumbi, Lucas Otieno, Danel Jones, Bernhard R Ogutu (2010). Impact of plasmodium falciparum infection in haematological parameters in children living in western Kenya. Malaria Journal.

[18] George OI, Ewelike-Ezeani CS (2011). Haematological changes in children with malaria infection in Nigeria. L Med Sci.

[19] Waitumbi JN, Opollo MO, Muga RO, Misore AO, Stoute JA (2000). Red cell surface changes and erythrophagocytosis in children with severe Plasmodium falciparum anemia. Blood.

[20] Xiang Ting Goh, Yvonne AL Lim, Indra Vythilingam, Ching Hoong Chew, Ping Chin Lee, Romano Ngui, Tian Chye Tan, Nan Jiun Yap, Veeranoot Nissapatorn, Kek Heng Chua (2013). Increased detection of Plasmodium knowlesi in Sandakan division, Sabah as revealed by PlasmoNex. Malaria Journal.

[21] Agravat, A H (2010). Hematological changes in pattentes of malaria. Journal of Cell and Tissue Research.

[22] Mepherson, R.A, Pincus,M.R (2007). Blood &Tissue Protozoa, Henry's Clinical Diagnosis &Management by Laboratory Methods.

